# 4-Acetylantroquinonol B Suppresses Prostate Cancer Growth and Angiogenesis via a VEGF/PI3K/ERK/mTOR-Dependent Signaling Pathway in Subcutaneous Xenograft and In Vivo Angiogenesis Models

**DOI:** 10.3390/ijms23031446

**Published:** 2022-01-27

**Authors:** Tur-Fu Huang, Shih-Wei Wang, Yu-Wei Lai, Shih-Chia Liu, Yu-Jen Chen, Thomas Y. Hsueh, Chih-Chung Lin, Chun-Hsuan Lin, Ching-Hu Chung

**Affiliations:** 1 Institute of Pharmacology, College of Medicine, National Taiwan University, Taipei 100, Taiwan; turfu@ntu.edu.tw; 2 Department of Medicine, Mackay Medical College, New Taipei City 252, Taiwan; shihwei@mmc.edu.tw (S.-W.W.); kumar1119@yahoo.com.tw (C.-H.L.); 3 Institute of Biomedical Sciences, Mackay Medical College, New Taipei City 252, Taiwan; 4 Division of Urology, Taipei City Hospital Renai Branch, Taipei 103, Taiwan; DAI77@tpech.gov.tw (Y.-W.L.); DAJ53@tpech.gov.tw (T.Y.H.); 5 Department of Urology, College of Medicine, National Yang Ming Chiao Tung University, Taipei 112, Taiwan; 6 Shu-Tien Urological Research Center, National Yang Ming Chiao Tung University, Taipei 112, Taiwan; 7 Department of Orthopaedics, MacKay Memorial Hospital, Taipei 104, Taiwan; jasonscliu649@gmail.com; 8 Department of Radiation Oncology, MacKay Memorial Hospital, Taipei 104, Taiwan; chenmdphd@gmail.com; 9 Department of Pharmacy, Taichung Veterans General Hospital Puli Branch, Nantou 545, Taiwan; bluereposeful@hotmail.com

**Keywords:** prostate cancer, *Antrodia cinnamomea*, 4AAQB, metastasis, VEGF, angiogenesis

## Abstract

Prostate cancer is a major cause of cancer-related mortality in men in developed countries. The compound, 4-acetylantroquinonol B (4AAQB), is isolated from *Antrodia cinnamomea* (commonly known as Niu-Chang-Chih), which has been shown to inhibit cancer growth. However, the anticancer activity of 4AAQB has not previously been examined in prostate cancer. This study aimed to investigate the effect of 4AAQB on cancer and angiogenesis, as well as to explore its mechanism of action. Human prostate cancer cells (PC3) and human umbilical vein endothelial cells (HUVEC) were used in cell viability, cell migration, and cell cycle functional assays to evaluate the anticancer and antiangiogenic efficacy of 4AAQB in vitro. The effects of 4AAQB in vivo were determined using xenograft and angiogenesis models. The signaling events downstream of 4AAQB were also examined. The 4AAQB compound inhibited PC3 cell growth and migration, and reduced in vivo cancer growth, as shown in a subcutaneous xenograft model. Furthermore, 4AAQB inhibited HUVEC migration, tube formation, and aortic ring sprouting; it also reduced neovascularization in a Matrigel implant angiogenesis assay in vivo. The 4AAQB compound also decreased metastasis in the PC3 prostate cancer model in vivo. Serum or vascular endothelial growth factor (VEGF)-induced VEGF receptor 2 (VEGFR2), phosphoinositide 3-kinase (PI3K)/Ak strain transforming (Akt), and extracellular signal-regulated kinase ½ (ERK ½) phosphorylation were attenuated by 4AAQB in both PC3 and HUVEC. In conclusion, 4AAQB is a potential candidate for prostate cancer therapy.

## 1. Introduction

Prostate cancer is one of the most common malignancies worldwide and has a high mortality rate, with more than one million deaths a year. It is frequently diagnosed in developed countries, especially in Europe and the United States, but the incidence of prostate cancer has increased in Asia, including Taiwan, over the past decade [1]. Prostate cancer-related deaths occur primarily due to the relapse of the disease and/or prostate cancer metastasis and invasion following initial therapy [2]. Hormone-related therapy and chemotherapies are the current options for prostate cancer treatment [3]. Because VEGF-A is overexpressed in prostate cancer and is associated with poor prognosis, these findings suggest that angiogenesis is important in prostate cancer and that antiangiogenic therapy may be an effective treatment option for prostate cancer. [4].

Angiogenesis is a physiological process essential for embryonic development and tissue repair and represents the formation of new blood vessels sprouting from the pre-existing vasculature. It is also considered to be one of the hallmarks of cancer initiation, progression, invasion, and metastasis, since cancer growth is believed to be angiogenesis-dependent [5]. A previous study demonstrated that angiogenesis is important for prostate cancer progression and metastasis [6]. Therefore, novel and improved therapies, which can effectively control prostate cancer cell migration, invasion, and cancer-associated angiogenesis, are required to prevent cancer progression and increase the survival of patients diagnosed with prostate cancer. VEGFR2 is the primary receptor for VEGF-A stimulation and angiogenesis mediation, in part through the activation of the ERK1/2 and Akt signal transduction pathways [7]. Studies have shown that the inhibition of VEGF/PI3K/mammalian targets of rapamycin (mTOR)/ERK signaling reduces angiogenesis, cancer growth, and metastasis [8,9].

*Antrodia cinnamomea* (*A. cinnamomea* also called *Taiwanofungus camphoratus*, *Antrodia amphorate*, *Ganoderma comphoratum*, and *Niu-Chang-Chih*) is a herb found in Taiwan, used in traditional medicines for diarrhea, hypertension, and cancer therapy [10]. Previous studies have shown that A. cinnamomea possesses anticancer, anti-inflammatory, and hepatoprotective properties [11,12,13]. Recently, this herb was demonstrated to have therapeutic effects against liver and breast cancers [14,15]. A compound isolated from the mycelium of this plant, 4AAQB, has been demonstrated to inhibit the growth of hepatocellular carcinoma cells [16]. Furthermore, our previous study found that 4AAQB inhibited hepatoma growth through a translation-dependent signaling pathway blockade and the inhibition of VEGF expression in vitro and in vivo [14]. However, the effects of 4AAQB and its derivatives on prostate cancer growth inhibition and antiangiogenesis have not been examined in detail. Thus, the mechanisms underlying the anticancer, antiangiogenic, and antimetastatic effects of 4AAQB were analyzed in this study.

## 2. Results

### 2.1. The Effects of 4AAQB on the Proliferation and Cell Cycle of Prostate Cancer Cells

**Figure 1:**
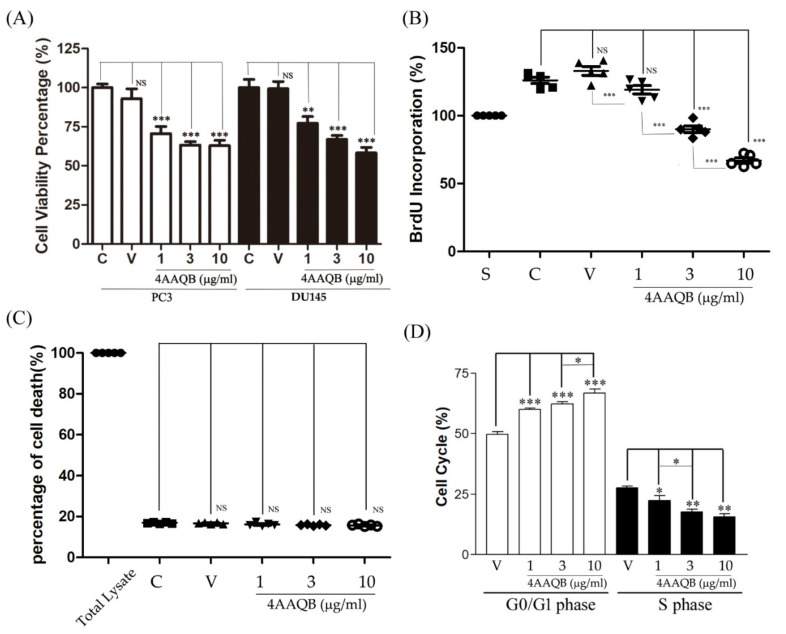
The effects of 4AAQB on prostate cancer cell viability, cytotoxicity, and cell cycle: (**A**) PC3 cell viability was detected via an MTT assay and DU145 cell viability was detected via an SRB assay; (**B**) the antiproliferation effect of 4AAQB in PC3 cell was detected via a BrdU assay. Data are represented as means ± SEM (*n* = 5); (**C**) LDH release was detected according to the manufacturer’s protocol. Data are represented as means ± SEM (*n* = 6); (**D**) PC3 cells were treated with a vehicle (V) or 4AAQB and analyzed by flow cytometry. The cell cycle distribution is shown as percentages in the cell cycle phases. Data are represented as means ± SEM (*n* = 6). * *p* < 0.05, ** *p* < 0.01, and *** *p* < 0.001 compared to the control (C) or vehicle (V) groups. S indicates the starvation group. NS: non-significance.

The effect of 4AAQB on prostate cancer cell proliferation was first determined by two prostate cancer cell lines (PC3 and DU145). As shown in Figure 1A, 4AAQB inhibited the viability of PC3 cells (as examined by an MTT (3-[4,5-dimethylthiazol-2-yl]-2,5 diphenyl tetrazolium bromide) assay) and DU145 cells (as examined by a sulforhodamine B (SRB) assay). A BrdU (5-bromo-2′-deoxyuridine) assay also demonstrated that 4AAQB treatment led to a significant inhibition of PC3 cell proliferation (Figure 1B). A LDH (lactate dehydrogenase) release test showed that 4AAQB was not cytotoxic toward PC3 cells (Figure 1C). Furthermore, 4AAQB also increased the proportion of cells in the G0/G1 phase and decreased those in the S phase in PC3 cells (Figure 1D).

### 2.2. 4 AAQB Inhibited PC3 Cell Migration, VEGF Production, and VEGFR2/Akt/mTOR/ERK1/2 Activation

**Figure 2:**
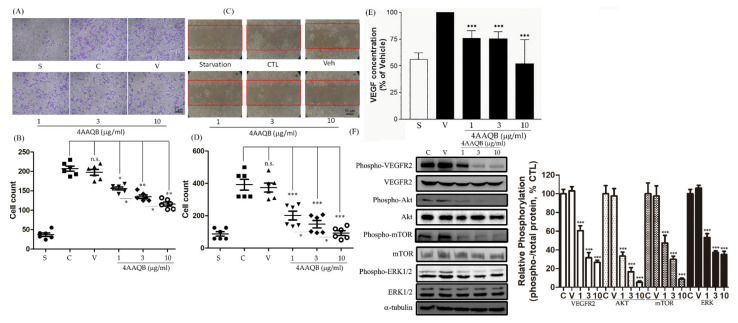
The compound, 4AAQB, inhibits PC3 cell migration, VEGF production, and signaling pathway proteins phosphorylation. (**A**) Migrated PC3 cells in the underside filter membrane were quantified (three fields per filter) by phase-contrast light microscopy. Data are represented as means ± SEM (*n* = 6). The cell number were calculated and are shown in (**B**); (**C**) wound-healing migration of PC3 cell monolayers treated with the vehicle or various doses of 4AAQB for 24 h. Data are represented as means ±SEM (*n* = 6). The cell numbers were calculated and are shown in (**D**); (**E**) VEGF protein expression was evaluated by ELISA and data are represented as means ± SEM (*n* = 6); (**F**) the PC3 cells’ protein phosphorylation was detected by Western blotting. Relative phosphorylation of protein is presented as mean density for the ratio between phosphorylated protein and total protein as determined by ImageJ. Data are represented as means ± SEM (*n* = 3). * *p* < 0.05, ** *p* < 0.01, and *** *p* < 0.001 compared to the control (C) or vehicle (V) groups. S indicates the starvation group. NS: non-significance.

The migration assays were performed using a modified Boyden chamber and wound-healing models. Migrated PC3 cells were observed in the control group, whereas 4AAQB treatment attenuated PC3 cell migration (Figure 2A). The quantification of the migratory cells is shown in Figure 2B. The cell migration in the wound-healing model also decreased in the 4AAQB-treated group when compared to the control (Figure 2C). The quantification of the migratory cells is shown in Figure 2D. The effect of 4AAQB on VEGF expression was tested. Moreover, 4AAQB inhibited VEGF secretion from PC3 cells (Figure 2E). The effect of 4AAQB on this signaling pathway was also monitored. The phosphorylation of VEGFR2/Akt/mTOR/ERK1/2 induced by 10% fetal bovine serum (FBS) was significantly inhibited by 4AAQB as compared to the control (Figure 2F). The quantification of the relative phosphorylation level of VEGFR2/Akt/mTOR/ERK1/2 is shown in Figure 2F.

### 2.3. 4 AAQB Showed Anticancer Effects in a Prostate Cancer Xenograft Model

**Figure 3:**
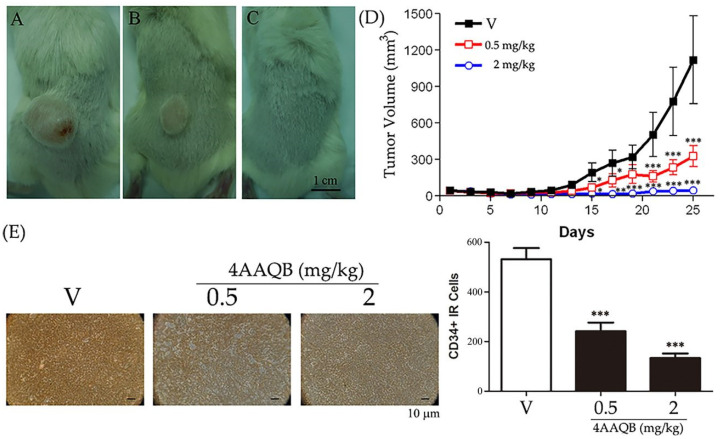
Anticancer effects of 4AAQB in in vivo models: SCID mice administered with the vehicle or indicated dosage of 4AAQB are displayed in (**A**–**C**) (vehicle (V), 0.5 mg/kg, and 2 mg/kg, respectively); the tumor volumes of the xenograft models are shown in (**D**); data are represented as means ± SEM (*n* = 8); (**E**) expression of CD34 in the cancer xenograft was revealed by staining, and the CD34-immunoreactive (IR) cells were counted. Data are represented as means ± SEM (*n* = 6). Scale bar = 1 cm (**A**–**C**) or 10 μm (**E**). * *p* < 0.05, ** *p* < 0.01, and *** *p* < 0.001 compared to the vehicle (V).

Mice treated by 4AAQB showed a decrease in PC3 cancer growth compared to that observed in the controls (Figure 3A–C: Vehicle, 0.5 mg/kg, and 2 mg/kg, respectively). The tumor volume was calculated and is shown in Figure 3D. Additionally, CD34 (an endothelial cell marker) expression was also decreased in the 4AAQB-treated group in comparison to that in the vehicle group (Figure 3E), revealing the antiangiogenic activities of 4AAQB.

### 2.4. 4 AAQB Inhibited HUVEC Proliferation and Migration

**Figure 4:**
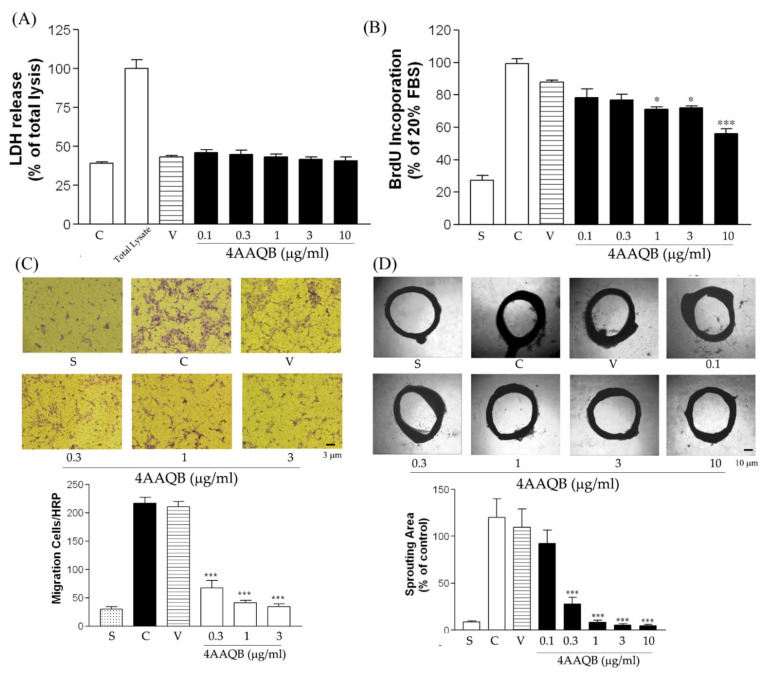
The effects of 4AAQB on HUVEC proliferation and in vitro and ex vivo migration: (**A**) LDH release was detected according to the manufacturer’s protocol. Data are represented as means ± SEM (*n* = 6); (**B**) HUVEC cell viability was detected by the BrdU assay. Data are represented as means ± SEM (*n* = 4); (**C**) migrated HUVEC in the underside filter membrane were quantified (three fields per filter) by phase-contrast light microscopy. Data are represented as means ± SEM (*n* = 6). The cell numbers were calculated; (**D**) rat aortic rings were photographed and the sprouting area was calculated and represented as mean ±SEM (*n* = 6). * *p* < 0.05 and *** *p* < 0.001 as compared to the control (C) groups. S indicates the starvation group.

The LHD release test results showed that 4AAQB was not cytotoxic toward HUVEC (Figure 4A). The BrdU assay demonstrated that 4AAQB treatment led to a significant inhibition of HUVEC proliferation (Figure 4B). An increase in migrated HUVEC was observed following stimulation with VEGF, while 4AAQB treatment was shown to attenuate HUVEC migration (Figure 4C). The ex vivo migration (aortic ring sprouting) induced by VEGF was also decreased in the 4AAQB-treated group (Figure 4D).

### 2.5. 4 AAQB Inhibited Tube Formation in Matrigel

**Figure 5:**
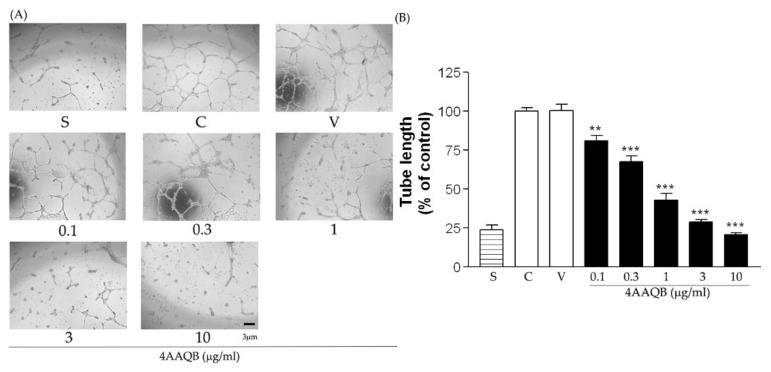
The effects of 4AAQB on HUVEC Matrigel tube formation. HUVEC treated with VEGF, with the vehicle (V), or various concentrations of 4AAQB were photographed (**A**), and the tube length were quantitatively analyzed as the fold change relative to the control group (**B**). Data are represented as means ± SEM (*n* = 6). ** *p* < 0.01 and *** *p* < 0.001 compared with the control (C) group.

HUVEC showed an increased number of branching tubes in Matrigel after incubation with VEGF, in comparison to that in the serum-free group, but 4AAQB treatment led to a decrease in these branching numbers (Figure 5).

### 2.6. 4 AAQB Inhibited Angiogenesis and Pulmonary Metastasis In Vivo

**Figure 6:**
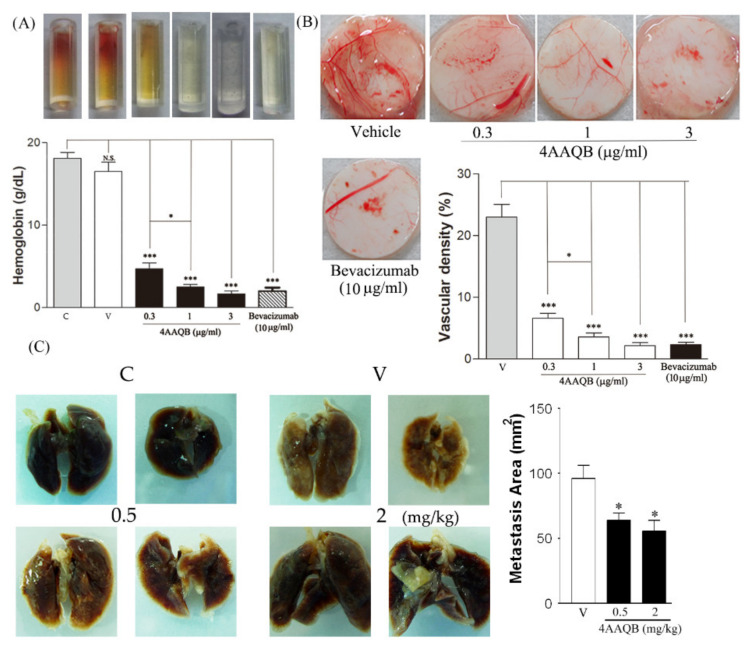
The effect of 4AAQB on the in vivo angiogenesis model. (**A**) DIVAA tubes were photographed and the hemoglobin level was measured. Data are represented as means ± SEM (*n* = 6); (**B**) the blood vascular densities of CAMs were calculated and is represented as mean ±SEM (*n* = 5); (**C**) the PC3 cells’ metastasis area of the lung surface is shown in white, and the background, in dark red. The white area was quantified using the ImageJ software. Data are represented as means ± SEM (*n* = 8). * *p* < 0.05 and *** *p* < 0.001 compared to the control (C) or vehicle (V) groups.

In the 4AAQB-treated group, lower red blood cell infiltration was observed in the implanted tubes, compared to those in the untreated mice (Figure 6A). The hemoglobin level was lower in the 4AAQB-treated mice as compared to control group. The chick embryo chorioallantoic membrane (CAM) assay was used to further examine the effect of 4AAQB on the formation of new capillaries; VEGF-induced neovascularization was almost abolished in the presence of 4AAQB in the CAM filter disk (Figure 6B). This neovascularization is also inhibited by Bevacizumab (positive control) (Figure 6A,B). Moreover, 21 days after a single intravenous bolus injection of PC3 cells, 4AAQB treatment resulted in a reduction in PC3 cell lung metastasis, compared to that in the untreated mice (Figure 6C).

### 2.7. 4 AAQB Inhibited VEGFR2/PI3K/mTOR/ERK1/2 Activation in HUVEC

**Figure 7:**
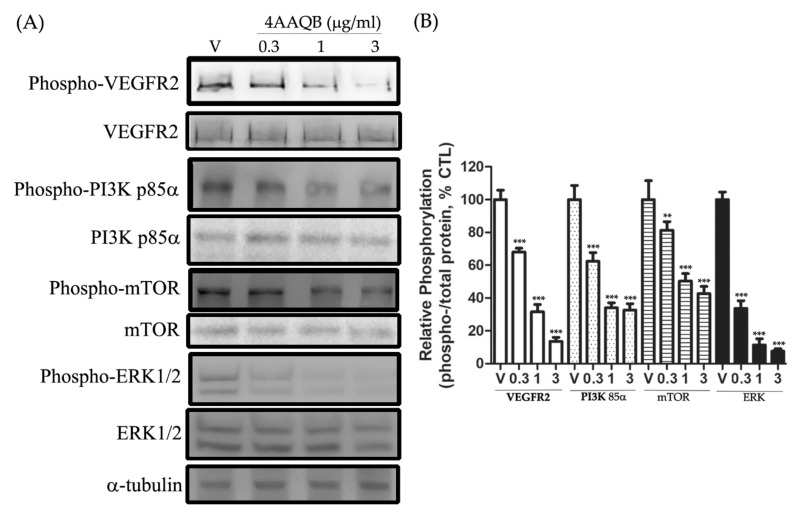
The effect of 4AAQB on VEGF-R2/PI3 p85α/mTOR/ERK pathways: (**A**) The inhibition effect of various concentrations of 4AAQB on the VEGFR2, PI3K, mTOR, and ERK pathways in HUVEC were detected by Western blotting; (**B**) relative phosphorylation of protein is presented as mean density for the ratio between phosphorylated protein and total protein as determined by ImageJ and the data are represented as means ± SEM (*n* = 5). ** *p* < 0.01 and *** *p* < 0.001 compared to the vehicle (V) group.

VEGF-induced VEGFR2, PI3K p85α, and mTOR phosphorylation was inhibited by 4AAQB treatment (Figure 7A). Furthermore, HUVEC pre-treated with 4AAQB showed a decrease in ERK1/2 activation (Figure 7A). The quantification of the relative phosphorylation level is shown in Figure 7B.

## 3. Discussion

*A. cinnamomea* has long been used in traditional medicine in Taiwan without any reported adverse effects [17]. Nonetheless, its biological activities, especially its anti-inflammatory and anticancer properties, have not been well documented. Antroquinonol, isolated from the mycelia of *A. cinnamomea*, exhibits anticancer activity and has been shown to inhibit the growth of different malignant cells in vitro [18]. Furthermore, 4AAQB inhibits hepatoma cell proliferation by modulating cell cycle regulators [19]. In this study, 4AAQB treatment significantly suppressed HUVEC proliferation, migration, and tube formation in vivo. To the best of our knowledge, the antiangiogenic effects of 4AAQB in vivo have not been reported yet.

Although cell cycle arrest and antiproliferative effects are beneficial for prostate cancer treatment, the microenvironment surrounding cancer cells is critical for the development of prostate cancer [3,4]. Angiogenesis consists of a multi-step process that involves cell migration and the proliferation of the endothelium, resulting in the formation of new vascular cells [20]. VEGF acts on its receptor, tyrosine kinase, at the cell surface, thereby regulating angiogenesis [21]. VEGF is important not only for mediating disease progression, but also because it plays a major role in prostate cancer metastasis [22]. Prostate cancer shows high VEGF-A expression [23]. Targeted antiangiogenic therapy for prostate cancer patients with either no metastases or limited, asymptomatic metastases, has resulted in improvements in relapse-free survival [24]. Therapies with dual-targeting effects, designed to inhibit both cancer cell growth and endothelial cell angiogenesis, may provide better outcomes for prostate cancer treatment. Herein, 4AAQB effectively inhibited AKT phosphorylation in PC3 cells (Figure 2). AKT pathway activity is important for the physiological processes in cells but also during the development of human cancers [25]. Aberrant activation of the AKT signaling pathway has been observed in various human cancers, and therefore, this pathway has become an attractive therapeutic target for anticancer-drug development. Dysregulated phosphorylation and tensin homolog cancer suppressor gene (PTEN) expression has frequently been observed in prostate cancer, and this dysregulation induces the aberrant activation of AKT pathway members, including mTOR [26]. These results indicate that the AKT pathway may be effectively targeted to inhibit the tumorigenicity of prostate cancer cells. Due to the modulation of the AKT pathway in prostate cancer, VEGF expression was also regulated, which implicates the regulation of angiogenesis [27,28]. Given that 4AAQB also inhibited VEGF expression (Figure 2E), this compound may become a good candidate for prostate cancer treatment.

The binding of VEGF to VEGFR2 leads to the activation of downstream signaling pathways, which results in increased cell proliferation [29]. VEGFR-2 activates many signal-transduction networks, such as focal adhesion kinase (FAK), PI3K/AKT, ERK1/2, SRC, and PLC-γ, thereby regulating angiogenesis-related processes [30]. Herein, VEGF-induced PI3K and ERK1/2 activation were considerably inhibited by 4AAQB pre-treatment, as was VEGF-induced VEGFR2 phosphorylation. This suggests that 4AAQB attenuates PI3K/AKT and ERK1/2 phosphorylation through a reduction in VEGF-receptor activation, contributing to the inhibition of angiogenesis.

Hormonal therapy is the basic treatment used for androgen-responsive metastatic prostate cancers. However, some patients do not respond to hormonal manipulations, and these patients are classified as having castration-resistant prostate cancer (CRPC). The chemotherapy options for CRPC, including cabiztaxel, docetaxel, mitoxantrone, and estramustine, were approved based on the survival benefit demonstrated in randomized studies [31]. Not only chemotherapies, but also immune checkpoint inhibitors, immunotherapy or targeted therapies have been approved for prostate cancer [3]. Angiogenesis is a key factor in prostate cancer progression, and microvessel density has been used to determine the prognosis of prostate cancer metastasis/survival. Therefore, angiogenesis inhibitors may also be useful for improving the therapeutic efficacy in prostate cancer treatment [32]. Combination therapy has been demonstrated to effectively improve therapeutic outcomes for advanced cancers [33]. Due to androgen signaling, which plays an important role in prostate cancer development and progression, the major treatment of prostate cancer is focused on targeting the androgen signaling pathway. However, there are not so many studies focused on combination therapy for the treatment of prostate cancer in the past decades. In this study, 4AAQB was shown to inhibit cell proliferation, migration, and tube formation in vitro, together with the inhibition of angiogenesis in the CAM model. The combination of 4AAQB with other chemotherapy or targeted therapies may provide an optional strategy for increasing the efficacy of prostate cancer therapy. However, this issue needs further investigation in the future.

## 4. Materials and Methods

### 4.1. Plant Material

*A. cinnamomea* was obtained from the Bioresources Collection and Research Center (Hsinchu, Taiwan; No. BCRC35716). A voucher specimen (MMC-002) was deposited in the Department of Medicine, Mackay Medical College (New Taipei City, Taiwan). The compound, 4AAQB, was isolated from the mycelium of *A. cinnamomea* through a fermentation process, including mycelial ethanol extraction, silica gel chromatography, and high-performance liquid chromatography purification (molecular weight of 462.6 g/mol) [16]. The structure of 4AAQB was identified by nuclear magnetic resonance (NMR) imaging (AMX-400, Bruker, Billerica, MA, USA) [34].

### 4.2. Chemical Materials

Fetal bovine serum was purchased from Gibco (Grand Island, NY, USA). Recombinant human VEGF was purchased from R&D Systems (Minneapolis, MN, USA), and MTT was obtained from Sigma (St. Louis, MO, USA). Matrigel was purchased from BD Bioscience (San Jose, CA, USA). LDH assay kits were purchased from Promega (Madison, WI, USA). Drabkin’s reagent kit 525 was obtained from Sigma (St. Louis, MO, USA). Endothelial cell growth supplement (ECGS) was obtained from Upstate Biotechnology (Lake Placid, NY, USA). Anti-phospho-VEGFR2 rabbit pAb and Anti-VEGFR2 rabbit pAb was purchased from Cell Signaling (Danvers, MA, USA). Furthermore, p-ERK1/2 (Tyr 204) rabbit pAb, ERK1/2 mouse mAb, p-PI3 kinase p85α(Tyr 508) rabbit pAb, PI3 kinase p85α mouse mAb, p-AKT (Ser 473) mouse mAb, Akt mouse mAb, p-mTOR (Ser 2448) mouse mAb, mTOR mouse mAb,α-tubulin mouse mAb, and peroxidase-conjugated anti-mouse and anti-rabbit antibodies were purchased from Santa Cruz Biotechnology (Dallas, TX, USA).

### 4.3. Cell Culture

The human prostate cancer cell lines PC3 and DU145 were obtained from the American Type Culture Collection. PC3 and DU145 cells were cultured in DMEM and MEM containing 10% fetal bovine serum (Gibco) at 37 °C. The HUVEC were provided by the Resource Centre National Research Programme for Biopharmaceuticals, Taiwan, and cultured in M199 with 20% FBS and 30 μg/mL of ECGS at 37 °C in an atmosphere with 5% CO_2_. HUVEC of the second to fifth passages were used in this study.

### 4.4. Cell Viability Assay

The MTT assay was used to measure the PC3 cells viability [34]. PC3 cells were sub-cultured (24-well plates, 80% cell density) and starved with serum-free DMEM (48 h). Then, the PC3 cells were grown in DMEM (with 10% FBS) with or without 4AAQB (48 h). The cells were incubated with MTT for another 4 h, and then lysed using dimethyl sulfoxide to measure the 550 nm absorbance with an ELISA reader. The SRB assay was used to measure the DU145 cells’ viability [14]. DU145 cells were sub-cultured (96-well plates) and starved with serum-free MEM (24 h). Then, the DU145 cells were grown in MEM (with 10% FBS) with or without 4AAQB (48 h). After treatment, 100 μL of trichloroacetic acid (10% (*w*/*v*)) was added to each well and incubated at 4 °C for 1 h. After dried in the air, to each well was added 50 μL of SRB solution (0.4% *w*/*v* in 1% acetic acid) and the incubated for 5 min at room temperature. The plates were then washed with 1% acetic acid and then air dried. The residual bound SRB was solubilized with 100 μL of 10 mM Tris–base buffer (pH 10.5) and the 490 nm absorbance was measured with an ELISA reader.

### 4.5. Cytotoxicity Assay

The cytotoxic effects were determined using lactate dehydrogenase (LDH) assay kits (Bio Vision Inc., Milpitas, CA, USA), following the manufacturer’s instructions and descriptions from a previous study [35]. The cells were incubated with 4AAQB for 48 h, and the LDH release was determined based on the ratio of the medium-to-cell-lysate LDH activity. The absorbance at 490 nm was finally recorded. The control was taken as the basal condition of LDH release.

### 4.6. Bromodeoxyuridine (BrdU) Proliferation Assay

PC3 cells (1 × 10^4^ cells per well) or HUVEC (2 × 10^4^ cells/well) were plated onto 96-well plates for 24 h, starved for an additional 16 h, and then treated with or without 4AAQB for 48 h. Cell proliferation was determined using a BrdU Cell Proliferation Assay Kit (Chemicon, Temecula, CA, USA), according to the manufacturer’s instructions.

### 4.7. Cell Cycle and VEGF Detection

Cell cycle experiments were performed with PC3 cells treated with vehicle as a control. According to the manufacturer’s protocol (Apoptosis and Cell Proliferation Kit; BD Biosciences, San Jose, CA, USA). VEGF detection was based on a VEGF Human ELISA Kit (Novex, Invitrogen, Carlsbad, CA, USA), used according to the manufacturer’s instructions [14].

### 4.8. Migration Assay

The migration assay was performed as previously described [14]. The lower surfaces of the inserts were coated with 0.25% gelatin overnight, followed by the addition of 0.6 mL of DMEM with 10% FBS (for PC3 cells) or M199 with VEGF (20 ng/mL for HUVEC) in the lower chamber. PC3 cells (2 × 10^4^ cells/insert) or HUVEC (1 × 10^4^ cells/insert) were added to the upper chamber and incubated with or without 4AAQB (30 min pre-treatment) (each group contained six wells). The non-migrated cells in the upper chamber were removed using a cotton swab after 16 h of incubation. Migrated cells in the underside filter membrane were fixed, stained, and quantified (three fields per filter) by phase-contrast light microscopy under a high-power field (HPF; magnification, 100×).

### 4.9. Two-Dimensional Migration Scratch Assay

PC3 cells were seeded into six-well plates (5 × 10^4^/well, 80% cell density) for 24 h and starved for 48 h. The dishes were scratched in a straight line using a pipette tip (200 μL) and incubated with or without 4AAQB in DMEM (with 10% FBS) for 24 h. Images of the wounded areas were captured via a microscope.

### 4.10. Tube Formation Assay

The tube formation assay was performed as previously described [36]. Matrigel was diluted to 4 mg/mL with cold phosphate-buffered saline (PBS), and 130 μL/well of Matrigel was placed into 48 wells, which were then incubated at 37 °C for 30 min to form a gel layer. HUVEC (1.2 × 10^5^ cells) in serum-free M199 with or without 4AAQB in the presence of VEGF (20 ng/mL) were seeded into the wells and incubated at 37 °C for an additional 16 h (each group contained six wells). Following incubation, the cells were washed/fixed with glutaldehyde and observed via a microscope.

### 4.11. Aortic Ring Sprouting Assay

This assay was performed as previously described [37]. Aortic rings were harvested from Sprague–Dawley rats (8~10 weeks old) and placed in 24 wells (coated with 130 μL of Matrigel each), and 50 μL of Matrigel (VEGF (20 ng/mL) in combination with 4AAQB, or not) was added to form a 3D Matrigel. Sprouting endothelial cells were observed via a microscope on day 8. The sprouting area was calculated and is represented as the mean ± SEM (each group contained six wells).

### 4.12. Western Blotting

The cells were pre-treated with 4AAQB for 30 min and stimulated with 10% FBS (for PC3 cell) or 20 ng/mL of VEGF (for HUVEC) for 10 min. The cells were then washed with phosphate-buffered saline and lysed with lysis buffer (containing PMSF, Na_3_VO_4_, aprotinin, and leupeptin) to release the proteins. The proteins were denatured and separated on 10% SDS-PAGE gels. Following protein transfer to polyvinylidene difluoride membranes, they were blocked in Tris-buffered saline with Tween-20/bovine serum albumin 5% and incubated sequentially with primary/secondary antibodies (the dilution factor for the antibodies used in this study was 1:1000). The results were detected using ECL. A-tubulin was used as loading control.

### 4.13. Animals

Male C57BL/6 and NOD SCID mice were provided by BioLASCO Taiwan Co., Ltd, Taipei, Taiwan. Chick embryos were provided by the Animal Health Research Institute. The animal experimental protocols were approved by the Institutional Animal Care and Use Committee of the College of Medicine, Tzu Chi University, Taiwan (Approval Code: 101069, Approval Date: 2 January 2013).

### 4.14. Directed In Vivo Angiogenesis Assay (DIVAA)

The DIVAA was performed as described previously [38]. A subcutaneous pocket (5 × 10 mm) was created with a sterile hemostat in male C57BL/6 mice. Sterile silicone tubes were filled with Matrigel containing VEGF (200 ng/mL) in the presence or absence of 4AAQ, and these tubes were implanted into the dorsal flanks of the mice (each group contained six tubes in two independent experiments). Bevacizumab was used as positive control. The neovessel formation rate in these tubes was quantified by determining the hemoglobin levels in the Matrigel using a Drabkin’s Reagent Kit 525 (Sigma-Aldrich) after 15 days.

### 4.15. Chick Chorioallantoic Membrane (CAM) Angiogenesis Assay

The CAM assay was performed as previously described [32]. Chick embryos were opened to allow access to the filter-paper disk (13 mm; Minipore, Bedford, MA, USA) containing VEGF (200 ng/disk) with various doses of 4AAQB (20 μL) (each group contained five eggs). Bevacizumab was used as positive control. The CAM tissues under the disks were resected and photographed using a digital camera (Nikon, Japan) after two days of incubation.

### 4.16. Subcutaneous Xenograft Models

PC3 (2 × 10^6^) cells at a volume of 100 μL were injected subcutaneously into the right flanks of male NOD SCID mice. The mice were randomly administered the vehicle or 4AAQB at a 0.5 or 2 mg/kg dose (each group contained eight mice), once daily, via intraperitoneal injection after the developing cancer reached 2 mm in diameter (approximately nine days). The cancer’s size was measured every two days and calculated according to the following formula: V = (length × width^2^/2). In accordance with the rules of the Animal Ethics Committee, the experiments were stopped if/when an unbearable situation appeared in the animals.

### 4.17. Immunohistochemical Analyses

Immunohistochemical analyses were performed as described previously [14]. Paraffin-embedded cancer sections were incubated with 1% H_2_O_2_, washed three times, blocked with 10% BSA, incubated with CD34 monoclonal antibody at 4 °C overnight, incubated with the biotinylated anti-mouse IgG and with 0.2% ABC solution, treated with DAB for 15 min, mounted on slides, air-dried overnight, and then cover-slipped (each group contained six slices).

### 4.18. Metastasis Assay

The 4AAQB was intraperitoneally administered to mice every day, beginning three days before PC3 cell injection, until day 21 after PC3 cells were injected into the tail vein of NOD SCID mice. The mice were sacrificed, and their lungs were removed and fixed in 10% formaldehyde. The PC3-metastasis area on the lung surface was observed to be white on a dark red background. The white areas were quantified using the ImageJ software. ImageJ software was downloaded from https://imagej.nih.gov/ij/download.html (accessed on 7 June 2013).

### 4.19. Statistical Analysis

All the values are presented as means ± standard errors of the mean (SEM), and the differences between the groups were assessed using one-way analysis of variance (ANOVA). Tukey’s test was used as a post hoc test in this study. All the *p*-values < 0.05 were considered to be statistically significant.

## 5. Conclusions

**Figure 8:**
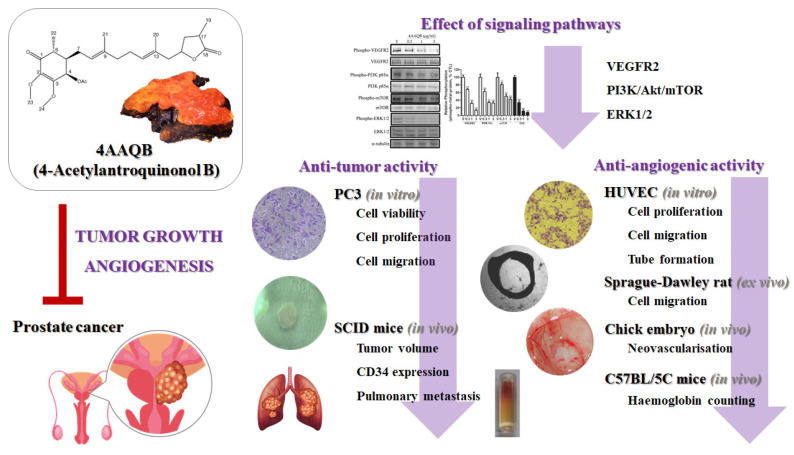
4AAQB inhibits cancer growth and angiogenesis via a VEGF/PI3K/mTOR/ERK-dependent signaling pathway.

In conclusion, this study demonstrated that 4AAQB inhibited both cancer growth and angiogenesis in vitro by inhibiting the activation of the VEGFR2, PI3K/Akt, mTOR, and ERK pathways. Additionally, 4AAQB exhibited anticancer activity in vivo, as demonstrated by the xenograft and pulmonary metastatic cancer models. These major results are summed up and shown in Figure 8. Collectively, our results suggest that 4AAQB may represent a novel anticancer agent for prostate cancer treatment. Further investigations using this compound alone or in combination with conventional anticancer drugs will help to further elucidate the effectiveness of this compound.

## Data Availability

Not applicable.
